# OBJECTIVE AND SUBJECTIVE SLEEP DURATION AND ACTIVITY LEVEL IN OLDER ADULTS WITH MILD DEMENTIA

**DOI:** 10.1093/geroni/igz038.1941

**Published:** 2019-11-08

**Authors:** Miranda V McPhillips, Jinyoung Kim, Darina V Petrovsky, Junxin Li, Sonia Talwar, Laurel Caffeé, Nancy Hodgson

**Affiliations:** 1 University of Pennsylvania, Philadelphia, Pennsylvania, United States; 2 John Hopkins University, Baltimore, Maryland, United States; 3 University of Pennsylvania, Philadelphia, United States

## Abstract

Little is known about the relationship between sleep duration and activities of daily living (ADLs) in those with mild dementia. We sought to examine the independent relationship between objective and subjective sleep duration and ADLs in community-dwelling older adults with mild dementia. Analyses were conducted on baseline data from participants enrolled in the Healthy Patterns Clinical Trial (Hodgson; R01NR015226). Measures included 24-hour wrist actigraphy for objective sleep duration, proxy-reported Pittsburgh Sleep Quality Index (sleep duration subscale) for subjective sleep duration and the Barthel Index for performance of ADLs. We used Spearman’s correlation and multivariate linear regression. A total of 30 individuals (56.7% male) aged 74.6 (SD 7.4) with mean Clinical Dementia Rating (CDR) scores of 1 (SD 0.5) were enrolled. Objective sleep duration ranged from 2.7 to 11.5 with mean 6.7 (SD 2.4) hours; subjective sleep duration ranged from 4 to 13.5 with mean 7.9 (SD 2.4) hours. Longer objective and subjective sleep duration were significantly associated with worse ADL scores (r = -0.48, p = 0.03; r = -0.59, p=0.007, respectively) in bivariate analyses. After controlling for age, CDR, and depression, subjective sleep duration was independently associated with ADLs (β = -1.90, p =0.03) and objective sleep duration trended toward significance (β = -1.47, p =0.10). These preliminary results suggest self-reported longer sleep could be indicative of declines in ADLs in older adults with mild dementia. Further prospective studies are necessary to determine the independent association between objectively assessed sleep duration and ADLs in patients with mild dementia.

